# The accumulation of vitrified oocytes is a strategy to increase the number of euploid available blastocysts for transfer after preimplantation genetic testing

**DOI:** 10.1007/s10815-016-0868-0

**Published:** 2017-01-09

**Authors:** Sandrine Chamayou, Maria Sicali, Carmelita Alecci, Carmen Ragolia, Annalisa Liprino, Daniela Nibali, Giorgia Storaci, Antonietta Cardea, Antonino Guglielmino

**Affiliations:** Unità di Medicina della Riproduzione—Centro HERA, via Barriera del Bosco n. 51/53, 95030 Sant’Agata Li Battiati, Catania Italy

**Keywords:** Aneuploidy, Next generation sequencing, Oocyte vitrification, Preimplantation genetic diagnosis, Preimplantation genetic screening

## Abstract

**Purpose:**

In a preimplantation genetic diagnosis for aneuploidy (PGD-A) program, the more embryos available for biopsy, consequently increases the chances of obtaining euploid embryos to transfer. The aim was to increase the number of viable euploid blastocysts in patients undergoing PGD-A using fresh oocytes together with previously accumulated vitrified oocytes.

**Methods:**

Sixty-nine patients with normal ovarian reserve underwent PGD-A for repeated implantation failure or recurrent pregnancy loss indication. After several cycles of ovarian stimulation, 591 accumulated vitrified oocytes and 463 fresh oocytes were micro-injected with the same partner’s semen sample. PGD-A was completed on 134 blastocysts from vitrified/warmed oocytes and 130 blastocysts from fresh oocytes.

**Results:**

A mean of 9.6% euploid blastocyst per micro-injected vitrified/warmed oocytes and 11.4% euploid blastocyst per micro-injected fresh oocyte were obtained (*p* > 0.05). The euploidy and aneuploidy rates were comparable in blastocysts obtained from micro-injected vitrified/warmed oocytes and fresh oocytes (42.5 versus 40.8% and 57.5 versus 59.2%, *p* > 0.05). Implantation rates of euploid blastocysts were comparable between the two sources of oocytes (56.0% from vitrified/warmed oocytes versus 60.9% from fresh oocytes, *p* > 0.05).

**Conclusions:**

Oocyte vitrification and warming do not generate aneuploidy in blastocysts. The number of viable euploid embryos for transfer can be increased by using accumulated vitrified oocytes together with fresh oocytes in ICSI.

**Trial registration:**

NCT02820415 ClinicalTrials.gov

## Introduction

In in vitro assisted reproduction, the failures in terms of clinical pregnancy and take home baby rate are mainly due to the transfer of embryos with undiagnosed aneuploidies. The correlation between the aneuploidy probability in the conceptus and the increasing maternal age has been well established [1]. Preimplantation genetic diagnosis for aneuploidy (PGD-A) relies on chromosomal profiling of embryos prior to implantation with the aim of transferring in utero only euploid embryos. PGD-A found a field of application in those patient groups with normal karyotypes and the lowest chance of take home baby rate. These patients are grouped in advanced maternal age (AMA), recurrent implantation failure (RIF), recurrent pregnancy loss (RPL), and severe male factor (SMF) [2].

Although widely practiced throughout the world, the efficacy of PGD-A was contested in 2007 because it was shown to decrease the success rates of in vitro fertilization [3, 4] when embryo biopsy was practiced on day 3 and chromosomal content was verified by fluorescent in situ hybridization. In current practice, the technical approaches of PGD-A have changed and overcome previous technical limitations. Advances in embryo culture make embryo biopsy applicable at the blastocyst stage (days 5–7); as a consequence, it is possible to biopsy a higher number of (trophectoderm) cells maintaining good embryonic implantation potential [5] and increasing the accuracy of genetic analysis [6, 7]. Several comprehensive chromosomal screening methods were applied for chromosomal diagnosis from single cells such as comparative genome hybridization array [8], single nucleotide polymorphism arrays with full molecular karyotyping [9, 10], qPCR that tests the chromosomes present based on few neutral amplifications per chromosome [11], and next-generation sequencing (NGS) where complete chromosomal content, enabling single gene disease and mitochondrial DNA mutations to be diagnosed at the same time [12, 13]. Encouraging results have been found from PGD-A application using these advanced technologies.

It is well known that the ovarian reserve, the ovarian response to gonadotrophin stimulation in terms of oocyte number and oocyte competence and the percentage of euploid embryos drastically decrease as maternal age increases. On the mean time, the more embryos available for biopsy consequently increases the chances of obtaining euploid embryos to transfer [14]. How can the number of blastocysts for biopsy on days 5–6 be increased considering that the chances of obtaining an euploid blastocyst to transfer are proportional to the number of available blastocysts for biopsy? Does an increased number of oocytes to micro-inject increase the number of euploid blastocysts to transfer?

In Italy, embryo accumulation by freezing is forbidden by law [15]. The only alternative to increase the number of blastocysts to diagnose is to increase the number of oocytes to microinject. This number can be increased by accumulating and vitrifying oocytes from repeated ovarian stimulations. In 2013, the practice committees of the ASRM and SART [16] established that oocyte vitrification followed by rapid warming [17] should no longer be considered as experimental as fertilization and pregnancy rates are similar to results from fresh oocytes in ICSI treatments. From a molecular point of view, it was found that mRNA content in oocyte after vitrification and warming is comparable with fresh oocytes [18] and the morphokinetic development of embryos from fresh and sibling vitrified/warmed oocytes are similar from the 2-cell stage [19]. To date, there have been no studies which show the efficacy of embryo development until blastocyst stage from vitrified/warmed oocytes compared to fresh oocytes from the same patients.

In the present study, we considered the hypothesis to accumulate vitrified oocytes with a view to increasing the number of oocytes for microinjection and consequently the number of blastocyst to diagnose for patients with normal ovarian reserve and candidate for PGD-A. They were proposed to undergo several cycles of ovarian stimulation. In the first cycles, mature (metaphase II) oocytes were vitrified and consequently accumulated. In the last cycle, the freshly produced mature oocytes and the previously accumulated ones were microinjected together with the same partner’s semen sample. The proportion of embryo development until blastocyst stage was compared between vitrified/warmed and fresh oocytes from the same patients. PGD-A was performed on blastocysts produced from the two sources of oocytes. The comprehensive chromosomal analysis of biopsied trophectoderm cells was performed using NGS technology. The number of available euploid blastocyst and the proportion of euploid/aneuploidy embryos vitrified/warmed from and fresh oocytes were compared. In conclusion, we evaluated the efficacy of oocyte accumulation by vitrification in increasing the number of available embryos for biopsy and the number of viable euploid embryos to transfer after PGD-A.

## Material and methods

**Fig. 1:**
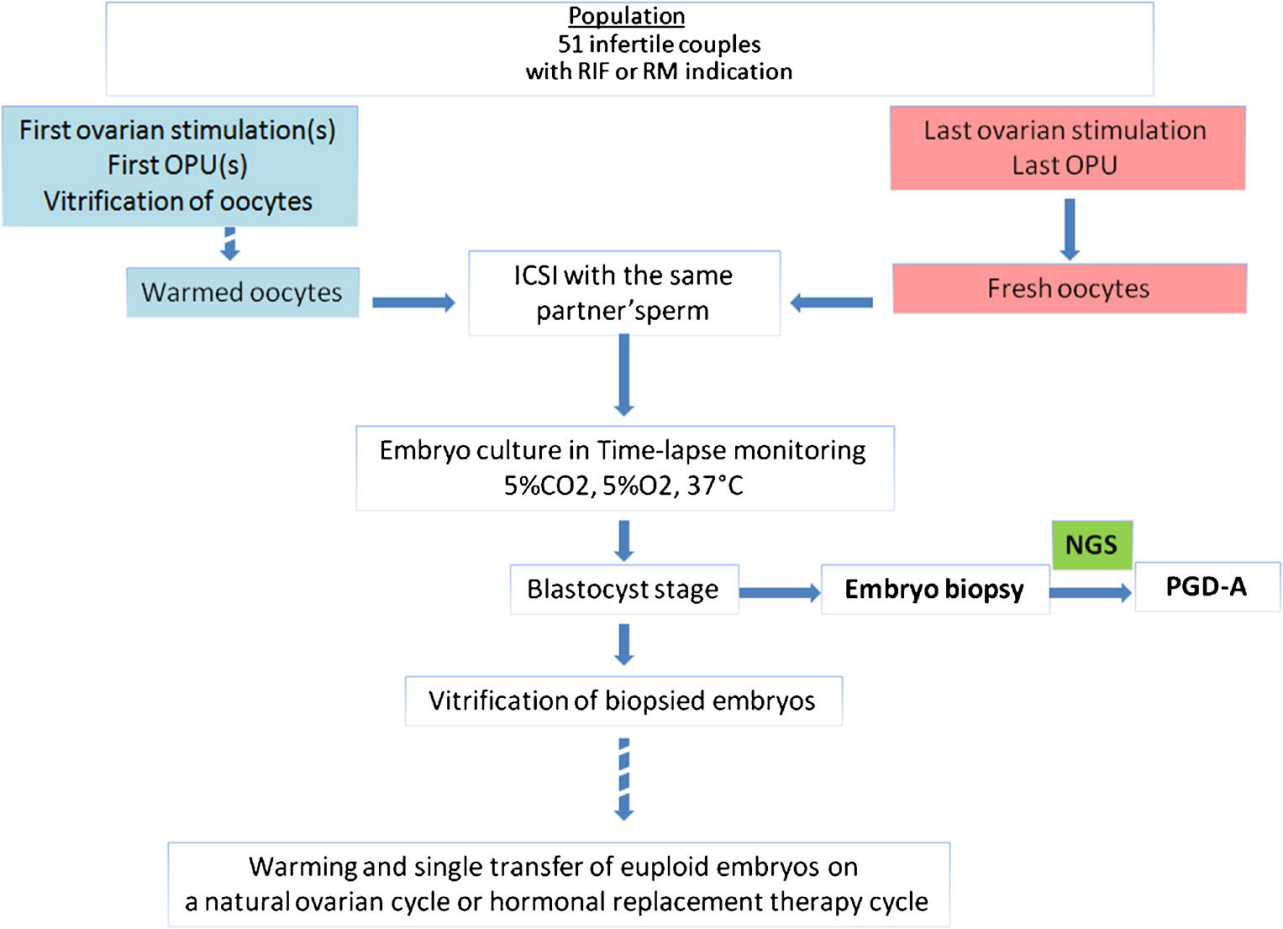
Protocol steps of PGD-A on blastocysts from vitrified/warmed and fresh oocytes

The materials and methods steps are illustrated in Fig. 1. Each step of the protocol and the sequence of each step have been approved by our Institutional Review Board. All participants gave written consent on all aspects of the study after having been informed. The present study was performed over a period of 25 months, from June 2014 to June 2016. The register number on www.clinicaltrials.gov was NCT02820415.

### Population and ovarian stimulation

The group was a compound of 69 patients aged between 29.0 and 42.3 years (mean age 36.6 years), with basal FSH on day 3 between 2.8 and 12.0 IU/l (mean 6.6 IU/l; 12.0 IU/l being the upper limit of normal FSH - Immulite 2000, Siemens-Germany). The indications for PGD-A were 47 patients for RIF according to Coughlan et al. [20] definition (transfer of at least four good-quality embryos in a minimum of three fresh or frozen cycles) and 22 patients for RM according to the definition of the Practice committee of ASRM [21] (two or more pregnancy losses). In each couple, the two partners had a normal karyotype. The patients underwent one to two cycles of ovarian stimulation to vitrify and accumulate oocytes and a last (second or third) cycle of ovarian stimulation. Ovarian stimulation was performed by the administration of recombinant FSH and LH (Gonal-F and Luveris: Merck-Serono, London, UK or Puregon, MSD, Franklin Lakes, USA) from cycle day 3. Initial doses were 225–300 IU/day for FSH and 75–150 IU/day for LH. Luteal gonadotrophin-releasing hormone antagonist was given when the leader follicle reached 14 mm in diameter with a dosage of 0.25 mg/day (Cetrotide : Merck-Serono, London, UK).

One ICSI was performed using the previously accumulated vitrified/warmed oocytes (the first or the first and second ovarian stimulations) together with the recently produced fresh oocytes (the second or third ovarian stimulation). The mean time between each cycle of oocyte vitrification and thawing to microinjection was 123 days (40–273 days).

At least 2 weeks before the first ovarian stimulation, the couples requested to know “the state of health of the embryos” to the clinical director of the centre as defined in comma 5, article 14 of the Italian law n. 40/2004 on Medically Assisted Procreation. At the same time, they signed informed consent forms on all procedures.

### Oocyte/embryo vitrification and warming

Oocyte vitrification commenced within 1 h of oocyte pick-up. The protocols used for oocyte [18] and embryo [25] vitrification and warming were previously described.

### ICSI on vitrified/warmed and fresh oocytes and embryo culture

Vaginal ultrasound-guided aspiration of oocyte−cumulus complex (OPU) was performed 35 h after human chorionic gonadotrophin administration (HCG 10,000 IU, Gonasi: AMSA, Rome, Italy). Oocyte denudation was performed 2 h after oocyte retrieval. ICSI was performed 1 h after oocyte denudation on fresh oocytes and 1 h after warming and in vitro culture on vitrified oocytes with the same sample of partner’s freshly ejaculated spermatozoa sample as previously described [22].

After ICSI, in vitro culture was carried out in 25 μl of continuous single culture complete medium with human serum albumin (Irvine Scientific, Santa Ana, USA) under mineral oil and in automated incubators with 5% CO2, 5% O2 at 37 °C, fitted with time-lapse imaging acquisition (Embryoscope, Unisense, Aarhus, Denmark). The entire embryo development has been followed and analyzed using morphokinetic parameters [23].

### Embryo biopsy

The biopsies were performed with an Olympus IX70 inverted microscope (UK) equipped with Hoffman optics and Narishige manipulators (USA). Two Narishige MMO-202D manipulators and two Narishige MM-88 micro-manipulators controlled two pipettes, each of which was attached to an IM-6 micro-injectors. Embryo biopsies were performed on expanded or in hatching blastocysts, grade 3 and 4 according to ASRM and ESHRE criteria [24]. The blastocyst was immobilized with a 120 um outer diameter holding micro-pipette in a 10 ul drop of HEPES buffered culture medium and under mineral oil. A few trophectoderm cells (5 to 10) were removed from a zona pellucida hole using a 1.48 um diode laser (OCTAX, Bruckberg, Germany) and a 20-um inner diameter biopsy pipette. After the biopsy procedure, each embryo was washed in culture medium and incubated until embryo vitrification and before blastocyst re-expansion. The biopsied trophectoderm cells were washed in sterile phosphate buffered saline (PBS) solution and transferred into a 0.2-ml Eppendorf tube containing 4ul of sterile PBS solution.

### Cell lysis, whole genome amplification, and NGS protocol

The biopsied trophectoderm cells were submitted to alkaline lyses and whole genome amplification according to Repli-g Single Cell protocol (Qiagen, Hilden, Germany). From this part of the protocol, all products and devices were from Life Technologies-Thermo Fisher (Carlsbad, USA). The whole amplified DNA was quantified by the Qubit 2.0 fluorometer and Qubit dsDNA HS assay kit. Libraries were prepared from 100 ng of each sample and barcoded with IonXpress Plus Fragment and IonXpress Barcode Adapter 1-32 or 1-16 kits. After quantification, each library was normalized to 100 pM according to the Ion Library Equalizer kit protocol. All libraries were mixed to obtain a final concentration of 8 pM and clonally amplified with the Ion PGM Template OT2 200 kit on the Ion OneTouch 2 System. Up to 20 enriched libraries were loaded on Chip 16 V2. DNA sequencing was performed on Ion PGM sequencing 200 kit on Ion Personal Genome Machine. The updated Torrent Suite Software was used for base calling and mapping on human genome reference sequence hg19. For each chromosome read coverage was corrected by guanine-cytosine calculation. Aneuploidy was diagnosed comparing data to baseline values multiple male samples. In all the process, a positive control with normal male DNA and a negative control from biopsy culture media were processed together with the samples to diagnose. Genetic analysis was validated when median absolute pairwise difference (MAPD) was inferior to 0.3. Chromosomal segments as short as 7 Mb could be detected.

The protocol was previously validated on single cells from amniocytes with the following karyotypes: 45,X0, 46,XX; 46,XY;47,XX,+21, 47,XY,+21, 47,XY,+13, 46,XY,dup(7)(q11.21q36.3), 46XY,del(9)(p24.3p13.1), 46,XY,dup(9)(p12q34.3), 46,XY,dup(13)(q11.2q24.3), 46XX,del(16)(p13.3p13.12), 46,XX,dup(16)(p13.12q24.3), 46,XY,del(2)(p25.3q24.3), 46,XX,dup(2)(q24.3q37.3), 46,XX,del(3)(q11.1q29), 46,XX,dup(4)(p16.3p35.2), 46,XX,dup(10)(p13.3q26.3), 46,XY,del(18)(p11.32p11.1), 46,XY,dup(18)(q11.1q23), 46,XY,dup(16)(p13.3q24.3).

### Endometrial preparation for embryo transfer

After warming, single blastocysts were cultured until blastocyst re-expansion. Single embryo transfers of warmed euploid blastocyts were performed on natural cycle at 7 days after LH surge or on day 5 of progesterone administration after E2 priming in an hormonal replacement therapy cycle.

### Statistical analysis

The statistical significances between the rates from vitrified/warmed and fresh oocytes were evaluated by z-test with *p* > 0.05 and 0.01.

## Results

### In vitro results

The results of oocyte vitrification, ICSI, embryo culture, and PGD-A analysis from vitrified/warmed and fresh oocytes are presented in Table 1.

**Table 1:** Results of oocyte vitrification/warming and ICSI, embryo culture, PGS-A analysis, and clinical outcomes from vitrified/warmed and fresh oocytes

	Vitrified/warmed oocytes (first ovarian stimulation)	Fresh oocytes (second ovarian stimulation)	*p*
N. patients	69	69	
N. ovarian cycles to accumulate oocytes	82	69	
In vitro results			
Metaphase II oocytes at OPU (mean per OPU)	615 (7.5)	463 (6.7)	
Vitrified oocytes	615	–	
Survived oocytes (survival rate)	591 (96.1)	–	
Micro-injected oocytes	591	463	
Zygotes (fertilization rate)	411 (69.5)	343 (74.1)	NS
Expanded/hatching biopsied Blastocysts (proportion on zygote)	138 (33.3)	135 (39.4)	NS
Vitrified biopsied blastocysts	138	135	
Genetic testing results			
Completed genetic analysis (percentage)	134 (97.1)	130 (96.3)	NS
N. euploid blastocysts (euploidy rate on completed genetic analysis)	57 (42.5)	53 (40.8)	NS
N. aneuploid blastocysts (aneuploidy rate on completed genetic analysis)	77 (57.5)	77 (59.2)	NS
Euploid/aneuploid blastocyst rate per initial MII oocytes	9.3/12.5	11.4/16.6	NS/0.05
Clinical outcomes			
Warmed, survived and transferred blastocysts	25	46	
Embryo transfers	25	46	
Clinical pregnancies (per embryo transfer)	15 (60.0)	30 (65.2)	NS
Implanted embryos (implantation rate)	14 (56.0)	28 (60.9)	NS
Arrested pregnancy	1	2	

*NS* non-significant

The patients produced a mean of 7.5 metaphase II oocytes that were vitrified during the first ovarian cycles and a mean of 6.7 metaphase II oocytes that were used as fresh. The survival rate of vitrified oocytes was 96.1% (591/615). The fertilization rate (411/591, 69.5% versus 343/463, 74.1%, *p* ˃ 0.05) was comparable between vitrified/warmed and fresh oocytes. The proportion of biopsied blastocysts calculated on MII oocytes at OPU was inferior in vitrified oocytes (138/591, 22.4%) compared to fresh oocytes (135/463, 29.2%, *p*˂0.01). Calculated on zygote number, these proportions (138/411, 33.6% versus 135/343, 39.4%, *p*˃0.05) were comparable between vitrified/warmed and fresh oocytes.

### Genetic testing results

The genetic analysis was validated and completed respectively in 97.1 and 96.3% of the biopsied blastocysts from vitrified/warmed and fresh oocytes (MAPD value superior to 0.3). More than 100,000 reads were produced per sample. After PGD-A, the euploid blastocyst rate was calculated on the number of MII oocytes at OPU and on the number of microinjected MII oocytes and compared between the group of vitrified/warmed oocytes with fresh oocytes. The euploid blastocyst rates were comparable in the two groups when calculated on MII oocytes at OPU (57/615, 9.3% from vitrifed/warmed oocytes versus 53/463, 11.4% from fresh oocytes, *p* > 0.05) and on microinjected MII oocytes (respectively 57/591, 9.6% and 53/463, 11.4%, *p* ˃ 0.05).

The euploid blastocyst rates calculated on the number of completed genetic diagnosis were comparable between the group of vitrified/warmed oocytes and the group of oocytes used as fresh (euploid blastocyst rate: 57/134, 42.5% from vitrified/warmed oocytes versus 53/130, 40.8% from fresh oocytes, *p*˃0.05).

### Clinical outcomes

All warmed euploid blastocysts (25 from vitrified/warmed and 46 from fresh oocytes) survived to warming and were transferred one at the time. The implantation rates were comparable between blastocysts from vitrified/warmed oocytes (14/25, 56.0%) and those from fresh oocytes (28/46, 60.9%, *p* ˃ 0.05). Forty-eight patients had euploid embryos from both sources of oocytes, 9 from only vitrified/warmed oocytes, 5 from only fresh oocytes. Seven patients had no euploid embryo from neither vitrified/warmed nor fresh oocytes.

To date, 34 children have been born 12 from vitrified/warmed oocytes and 22 from fresh oocytes. Eight pregnancies are on-going. All genetic analyses were confirmed by prenatal or postnatal genetic diagnosis.

## Discussion

We present the results of PGD-A from blastocysts obtained from accumulated vitrified/warmed and fresh oocytes from the same patients. Oocyte vitrification (and warming) leads to a similar rate of fertilization, and blastocyst calculated on zygote number. Despite 96.1% of oocyte survival rate to vitrification process, the percentage of biopsied blastocyst calculated on MII oocytes at OPU was inferior in vitrified oocytes (22.4%) compared to fresh oocytes (29.2%, *p* ˂ 0.01). Calculated on zygote number, the blastocyst rates were comparable between the two groups. The euploidy/aneuploidy rates in blastocysts calculated on MII oocytes at OPU or microinjected MII oocytes were comparable in the two sources of oocytes from the same patients. Implantation rates of warmed euploid blastocysts were comparable between the two sources of oocytes when transferred on natural cycles. Oocyte accumulation by vitrification increases the number of available embryos for biopsy and hence the number of viable euploid blastocysts to transfer, increasing the chances of obtaining a pregnancy.

After oocyte accumulation by vitrification and their addition to the fresh oocytes cohort, the number of expanded or in-hatching blastocyst available for biopsy nearly doubled (1.7 blastocyst per cycle with vitrified/warmed oocytes plus 2.0 blastocysts per cycle using fresh oocytes).

Milàn et al. [26] and Cobo et al. [27] first proposed oocyte accumulation by vitrification as a strategy to increase the number of oocytes for microinjection, increasing the chances of positive outcomes in AMA and low-responder patients. As Forman et al. [28], we found that oocyte vitrification does not increase the risk of embryo aneuploidy nor diminish the implantation potential. Two previous studies performed on a very limited number of cases provided good clinical results of preimplantation genetic analysis on vitrified blastocysts obtained from vitrified oocytes [29, 30]. Zygote and embryo banking are also alternatives to increase the number of embryos to biopsy and transfer [31]. In Italy, these strategies are not applicable because embryo freezing is only allowed when embryo transfer cannot be performed due to unexpected medical causes at the moment of fertilization. In 2009 and after the sentence the Constitutional Court embryo freezing became allowed to avoid multiple pregnancy risk due to the transfer of several embryos [32]. To freeze and accumulate embryos for other uses such as later diagnosis remains illegal. On the other hand, it is permitted to accumulate oocytes. At any time of ART treatment and before embryo transfer in utero, the couple can request to know “the state of health” of the embryos as written in the Italian law on Medically Assisted Procreation [15] and provided in our centre.

In the present study, comprehensive chromosomal analyses were performed on blastocysts from the same patients, after two or more ovarian stimulations with the same ovarian stimulation protocol and within a limited duration (4.2 months). For 48 couples (69.6%), euploid embryos were obtained from both vitrified/warmed and fresh oocytes. Seven couples (10.1%) had no euploid embryo from neither vitrified/warmed nor fresh oocytes. Nine couples (13.0%) had euploid embryos only from vitrified/warmed oocytes and 5 couples (7.2%) only from fresh oocytes. For these last 5 couples plus the 7 couples that had no euploid embryo from vitrified/warmed and fresh oocytes, oocyte accumulation gave no further advantage because no supplementary euploid embryo was obtained from additional ovarian stimulation (12/69, 17.4% of the couples). This aspect is a limitation of the oocyte accumulation strategy that is not predictable a priori. 89.9% of the patients undergoing the present protocol of oocyte accumulation had euploid blastocysts to transfer. This rate is higher than in Forman et al. study [45] where 67% of good responder patients reached embryo transfer with fresh euploid blastocysts and without oocyte accumulation. According to the IVF with PGD-A survey in the USA for the period 2011–2012 [37], this rate varied between 53.1% for patients over 37 years old and a maximum of 77.5% for patients under 35 years old. The present strategy of oocyte accumulation leaded to an increased proportion of patients reaching embryo transfer due to an increased number of available euploid blastocysts.

In terms of the cost for couples, the cost of diagnosis calculated on available blastocyst was decreased of one third when blastocysts from fresh and vitrified/thawed oocytes were biopsied compared to blastocysts from fresh oocytes only. No patient experienced particular discomfort as a result of multiple ovarian stimulation for oocyte accumulation. When no euploid blastocyst is available to transfer from successive ovarian stimulations, a second biopsy on a different trophectoderm spot could be proposed after embryo thawing to recover aneuploid/diploid blastocysts firstly diagnosed as aneuploid [33, 34].

The concept of chromosomal diagnosis on the embryo before transfer in a view to increase implantation rate in those patients with a poor prognosis (AMA, RIF, RM, SMF) has been globally practiced since the 1990s. Despite accumulated results and the yearly reports of the ESHRE PGD consortium, the study of Mastenbroek et al. [3] made the scientific community realise that the protocols used for embryo biopsy and genetic analysis applied at that time were not appropriate and decreased the chances of pregnancy [4]. Today, the situation has improved, the population that would benefit most from PGD-A in improving pregnancy rate decreasing miscarriage has been mainly demonstrated for young patients, with a good prognosis or normal ovarian reserve according to the reviews of Dahbouh et al. [35] and Lee et al. [36]. Conversely, the analysis of a large data set showed that PGD-A significantly decreased live-birth rates per transfer for the youngest group of patients [37].

Duplications, deletions, de novo abnormalities regarding entire chromosomes or small segmental chromosomal gain and loss [38] are diagnosable by using NGS coupled with single-gene analysis when necessary. Furthermore, NGS is the only technology that gives the possibility to quantify mitochondrial DNA that has recently appeared as an indicator of embryonic vitality [39], mosaicism that concerns 4.8% of the blastocysts with a percentage that can reach 50% of the trophectoderm cells [33, 40–42], de novo translocations in the embryo [6] and copy number variation sequencing associated with chromosome disease syndromes [43].

In 2013, ASRM and SART [16] declared that oocyte vitrification should not longer be considered as experimental for good prognosis patients. A consensus of experts agree that embryo development after oocyte vitrification should be the same for the comparable population of fresh embryos [44]. Nevertheless, very few studies have compared embryo development until blastocyst stage from fresh and vitrified/warmed oocytes and none from the same patients. According to our data, the blastocyst rate was statistically inferior in the vitrification group when calculated on micro-injected oocytes but comparable when calculated on zygote number. The results were obtained using vitrified/warmed and fresh oocytes from the same patients. In our clinic and from the results over the two last years, the clinical pregnancy rates (32.2% from vitrified/warmed oocytes versus 35.3% from fresh, *p* > 0.05) and miscarriage rates (5.4% from vitrified/warmed oocytes versus 12.4% from fresh, *p* > 0.05—data not published) were comparable in ICSI from fresh and vitrified/warmed oocytes. For this reason, oocyte accumulation by vitrification as a way to maximize the number of euploid embryos to transfer in PGD-A patients is actively promoted. From the present data, the equivalent euploid blastocyst rate obtained from fresh and vitrified/warmed oocytes from the same patients (mean age of 36.8) demonstrates that oocyte vitrification does not generate additional aneuploidy on blastocysts.

## Conclusion

The applicability of PGD-A on blastocyst relies on the number of embryos available for biopsy. From the present results, the euploidy and aneuploidy rates in blastocysts from vitrified/warmed and fresh oocytes from the same patients and within a mean period of 4 mouths were comparable. The present study demonstrates for the first time that oocyte vitrification/warming processes do not generate surplus aneuploidy in the blastocysts. The number of available blastocysts for biopsy and consequently the number of viable euploid blastocysts for transfer can be increased by accumulating oocytes to microinject produced from repeated ovarian stimulations. Warmed euploid blastocysts produced from vitrified oocytes have an implantation potential comparable to warmed euploid blastocysts produced from fresh oocytes. In the present, this strategy has been validated for patients with a normal ovarian reserve. The potentiality of this strategy remains to be established for patients with reduced ovarian reserve and in preimplantation genetic protocols.
